# Effect of Continuous Positive Airway Pressure on Symptoms and Prevalence of Insomnia in Patients With Obstructive Sleep Apnea: A Longitudinal Study

**DOI:** 10.3389/fpsyg.2021.691495

**Published:** 2021-07-22

**Authors:** Ragnhild Stokke Lundetræ, Ingvild West Saxvig, Harald Aurlien, Sverre Lehmann, Bjørn Bjorvatn

**Affiliations:** ^1^Department of Global Public Health and Primary Care, University of Bergen, Bergen, Norway; ^2^Centre for Sleep Medicine, Haukeland University Hospital, Bergen, Norway; ^3^Norwegian Competence Center for Sleep Disorders, Haukeland University Hospital, Bergen, Norway; ^4^Department of Clinical Neurophysiology, Haukeland University Hospital, Bergen, Norway; ^5^Department of Clinical Science, University of Bergen, Bergen, Norway

**Keywords:** CPAP, insomnia, COMISA, sleep apnoea, obstructive sleep apnea, CPAP adherence

## Abstract

**Objective:**

Obstructive sleep apnea (OSA) and insomnia are the two most common sleep disorders. Continuous positive airway pressure (CPAP) is considered first-line treatment for OSA. In the present study, we assess the effect of CPAP on symptoms and prevalence of insomnia in patients with OSA. We hypothesized a decrease in insomnia symptoms from CPAP initiation to follow-up, and that this decrease would depend on CPAP adherence.

**Materials and methods:**

The sample included 442 patients diagnosed with OSA [mean age 54.9 years (SD = 12.1), 74.4% males] who started treatment with CPAP at a university hospital. OSA was diagnosed according to standard respiratory polygraphy. Mean apnea-hypopnea index (AHI) was 30.1 (SD = 21.1) at baseline. Insomnia was assessed prior to CPAP treatment (baseline) and at follow-up after a median of 19.9 weeks (range 6–52 weeks) with the Bergen Insomnia Scale (BIS). CPAP adherence was defined as an average use of ≥ 4 h per night, whereas non-adherence was defined as < 4 h per night.

**Results:**

There was a significant decrease in BIS scores from baseline (mean = 18.8, SD = 9.8) to follow-up (mean = 12.9, SD = 9.9), *p* < 0.001. Cohen’s *d*(0.65) indicated a moderate effect size. The reduction in BIS scores was depending on CPAP adherence (interaction effect *F*(1,440) = 12.4, *p* < 0.001), with larger reduction in the adherent group than in the non-adherent group. The proportion of patients with chronic insomnia was significantly reduced from 51.1% at baseline to 33.0% at follow-up (*p* < 0.001).

**Conclusion:**

Overall, there was a significant reduction in insomnia symptoms from baseline to follow-up. The improvement was significant in both adherence groups, but the degree of improvement was larger among patients who were adherent to CPAP. Furthermore, there was a significant reduction in the prevalence of chronic insomnia at follow-up compared to baseline. This suggests that CPAP effectively reduces both the presence of insomnia and the severity of insomnia symptoms in some patients with OSA.

## Introduction

Obstructive sleep apnea (OSA) and insomnia are the two most common sleep disorders in the general population. OSA affects at least 8–10% whereas insomnia affects about 7–16% of the population, depending on the diagnostic criteria used (Ohayon and Reynolds, 2009; Hrubos-Strøm et al., 2011; Peppard et al., 2013; Pallesen et al., 2014). OSA is characterized by breathing pauses, oxygen desaturation, and arousals during sleep (American Academy of Sleep Medicine, 2014). In addition, symptoms such as snoring and daytime sleepiness are common. The apnea-hypopnea-index (AHI) is used to classify the severity of OSA, in which a higher AHI indicates more severe OSA. Chronic insomnia is characterized by self-reported difficulties initiating sleep, maintaining sleep, and/or early morning awakenings from sleep, which are associated with daytime impairment for a period of 3 months or more (American Psychiatric Association, 2013).

Comorbid insomnia and sleep apnea (COMISA) was first described by Guilleminault et al. (1973). Since then, several studies have described the co-occurrence of obstructive sleep apnea and insomnia, and the relation is most likely bidirectional (Sweetman et al., 2019a). Among insomnia patients, 29–67% fulfill the diagnostic criteria for OSA, whereas 39–58% of OSA patients report insomnia symptoms (Krakow et al., 2001; Luyster et al., 2010; Björnsdóttir et al., 2012; Bjorvatn et al., 2015).

The breathing pauses caused by OSA may cause, exacerbate, or contribute to insomnia by increasing the number of nightly awakenings. Continuous positive airway pressure (CPAP) is considered first line treatment for moderate to severe OSA and is effective in keeping the airways open and thereby reducing the apneas. However, the major problem with CPAP is adherence (Weaver and Grunstein, 2008). Comorbid insomnia can make it difficult to treat OSA with CPAP, as patients with chronic insomnia typically are more sensitive to disturbing factors at bedtime. Noise from the machine, leakage, and problems wearing the mask can lead to lower CPAP adherence. Several studies have reported poor CPAP adherence and early quitting of CPAP therapy among patients with insomnia symptoms (Wickwire et al., 2010; Eysteinsdottir et al., 2017). Philip et al. reported that CPAP adherence was negatively associated with both sleep onset and sleep maintenance insomnia symptoms, but not with early morning awakenings (Philip et al., 2018), whereas Glidewell et al. reported in a cohort study that both adherence to positive airway pressure (PAP) and OSA severity were predictors of insomnia improvement (Glidewell et al., 2014). Björnsdóttir et al. (2013) previously showed that CPAP treatment significantly reduced symptoms of insomnia. However, the insomnia definition was based on three questions from the Basic Nordic Sleep Questionnaire, and the authors called for studies having more detailed questions on insomnia. Accordingly, there is need for more studies using a validated questionnaire based on the standard criteria for the insomnia diagnosis.

Cognitive behavioral therapy for insomnia (CBTi) is considered treatment of choice for chronic insomnia (Riemann et al., 2017). There is emerging evidence that patients with COMISA should be treated in a multidisciplinary way with concurrent CPAP and CBTi (Sweetman A. et al., 2017; Bahr et al., 2018; Sweetman et al., 2019a; Ong et al., 2020b). However, there are no definitive guidelines for how to combine or integrate CPAP and CBTi in the case of COMISA. A recent randomized controlled trial by Ong et al. (2020a) among 121 patients reported a greater reduction in insomnia severity among patients receiving both CPAP and CBTi vs. CPAP alone. Sweetman et al. (2020) were the first to show that CBTi reduces OSA severity in a randomized controlled trial. Unfortunately, few therapists offer CBTi, and it is rarely offered in clinics focusing on OSA treatment. Morin et al. (2005) suggest that the reasons for this may be that CBTi is time consuming and not easily available. In a recent study, however, Bjorvatn et al. (2018a) did not find effect of a self-help book for insomnia in patients with COMISA. Thus, there is need for more studies on treatment interventions in clinical samples with COMISA. Furthermore, in a recent review, Sweetman et al. (2019a) suggest that future research should investigate the bidirectional relationships between OSA and insomnia, thereby examining the effect of CPAP therapy on manifestations and severity of insomnia symptoms.

Against this backdrop, we aimed to investigate the effect of CPAP on insomnia symptoms and prevalence of chronic insomnia in patients with OSA. We hypothesized a decrease in insomnia symptoms at follow-up, and that this decrease would depend on CPAP adherence. Furthermore, we hypothesized a reduced prevalence of chronic insomnia at follow-up compared to baseline.

## Materials and Methods

### Participants and Setting

The sample comprised 442 patients diagnosed with OSA and initiating treatment with CPAP (Airsense^TM^ 10 Autoset^TM^ or S9 AutoSet^TM^, ResMed Norway AS) at a Norwegian University hospital between 2011 and 2018. The patients were referred with suspicion of OSA from a large geographic area in Western Norway. All patients in this region of Norway receive CPAP from this hospital. In total, 329 (74.4%) of the patients were males and 113 (25.6%) were females. Mean age was 54.9 ± 12.1 years (range 21–82).

### Study Design

**FIGURE 1:**
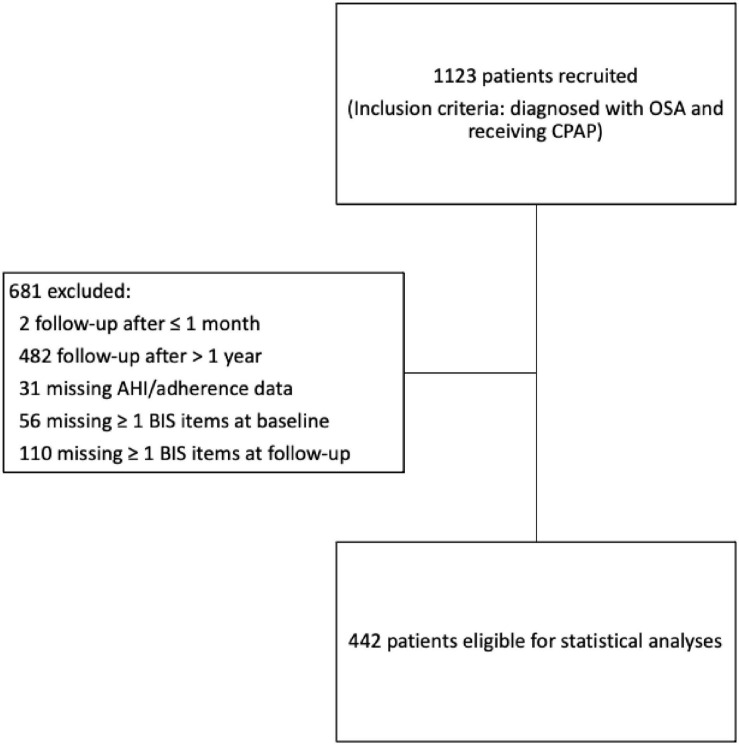
Overview of the participants in the study.

In this longitudinal study, inclusion criteria were AHI ≥ 5, receiving CPAP, and answering all six items on Bergen Insomnia Scale (BIS) at baseline and at follow-up. Exclusion criteria were attending follow-up in less than 1 month or more than 1 year after baseline, missing one or more items on Bergen Insomnia Scale (BIS) at baseline and/or follow-up, and missing AHI and/or adherence data measured from the CPAP machine at follow-up (Figure 1).

### Instruments

#### Polygraphy

All patients were diagnosed with OSA based on standard out-of-center respiratory polygraphy using a type 3 portable monitor (Embletta^TM^ or NOX T3, Resmed Norway AS) as described in previous publications (Bjorvatn et al., 2015). The scoring criteria from the 2007 manual of the American Academy of Sleep Medicine (Iber et al., 2007) were used. Thus, apneas were defined as a reduction of 90% or more of baseline nasal airflow with a duration of at least 10 s. Hypopneas were defined as a nasal flow reduction of 30–90% of baseline, lasting at least 10 s accompanied by an oxygen desaturation of ≥ 4%. The severity of obstructive sleep apnea was classified in accordance with the apnea hypopnea index (AHI): mild OSA (AHI 5–14.9); moderate OSA (AHI 15–29.9); severe OSA (AHI ≥ 30). In addition, for mild OSA, presence of symptoms or a relevant comorbidity, such as hypertension or diabetes mellitus, had to be present. Mean baseline AHI in the current sample was 30.1 ± 21.1 (range 5.0–135.2).

#### CPAP

Adherence was categorized at follow-up based on the average CPAP use the last 90 nights as measured objectively by the CPAP device. Patients using the CPAP ≥ 4 h per night were defined as adherent, whereas patients using the CPAP < 4 h per night were defined as non-adherent (Povitz et al., 2014; Yu et al., 2017; Zheng et al., 2019). AHI at follow-up was measured objectively by the CPAP device.

#### Questionnaires

Prior to CPAP treatment and at follow-up, which was scheduled after about 3 months, the patients completed a questionnaire including questions about age, sex, marital status, and diagnosed hypertension, diabetes mellitus, angina pectoris, myocardial infarction, or stroke. Anxiety and depression were assessed with the validated Hospital Anxiety and Depression Scale (HADS) (Zigmond and Snaith, 1983). The scale consists of 14 separate statements, divided into two seven-item subscales, one for anxiety (HADS-A) and one for depression (HADS-D). Each statement has four response alternatives, ranging from a healthy state (0) to maximum symptom severity (3). The composite HADS score for either anxiety or depression is consequently ranging from 0–21. A score of 11 or higher on each subscale was used to indicate probable anxiety and depression cases (Zigmond and Snaith, 1983; Bjelland et al., 2002). In addition, weight and height were measured, and body mass index calculated (kg/m^2^). To assess insomnia symptoms, the patients completed the validated Bergen Insomnia Scale (BIS) both at baseline and at follow-up (Pallesen et al., 2008). BIS consists of six items and was developed based on the diagnostic criteria for insomnia according Diagnostic and Statistical Manual of Mental Disorders, 4th Edition, Text Revision (DSM-IV-TR) (American Psychiatric Association, 2000). The items are scored along an eight-point scale indicating the number of days per week (0–7 days) for which a specific insomnia symptom is experienced. Until 2018, BIS addressed symptoms during the past month, whereas from 2018 the scale addressed symptoms during the past 3 months, in line with the updated criteria for chronic insomnia (DSM-5). A total of 73 among the 442 patients answered BIS regarding the past 3 months. The first four items refer to sleep onset (sleep latency exceeding 30 min), wake after sleep onset (more than 30 min), early morning awakening (more than 30 min), and non-restorative sleep, whereas the two latter items refer to daytime impairment and dissatisfaction with sleep. The scale is used as a continuous scale (values 0–42), where higher scores indicate greater degree of insomnia. Chronic insomnia, according to the updated diagnostic criteria in International Classification of Sleep Disorders, version 3, and the Diagnostic and Statistical Manual of Mental Disorders, 5th edition, was defined as scoring ≥ 3 days per week on at least one of the first three items as well as ≥ 3 days per week on at least one of the two latter items (American Psychiatric Association, 2013; American Academy of Sleep Medicine, 2014). Non-restorative sleep was previously part of the diagnostic criteria for chronic insomnia (American Psychiatric Association, 2000), but is no longer included according to the fifth and latest version of the Diagnostic and Statistical Manual for Mental disorders (DSM-5) and the International Classification of Sleep Disorders-3 (ICSD-3). Thus, the question regarding non-restorative sleep was only included when calculating total BIS score. The prevalence of insomnia subtypes at baseline among the patients with chronic insomnia was assessed according to the DSM-5 criteria. Those who scored ≥ 3 days on one of the first three questions of BIS (sleep onset latency, wake after sleep onset, and early morning awakening) in addition to ≥ 3 days on question five and/or six (daytime impairment and dissatisfaction with sleep) were classified as having sleep onset, sleep maintenance and early morning awakening insomnia subtypes, respectively.

### Ethics

The Regional Committee for Medical and Health Research Ethics, Health Region West (REC 2018/337) approved the study. All patients provided written informed consent.

### Statistical Methods

The Statistical Package for the Social Sciences (SPSS) version 26.0 was used for the data analyses. Paired-samples *t* tests were conducted to compare BIS scores at baseline and follow-up. Normality checks were carried out, and the assumptions met. Paired-samples *t* tests were used to perform subsequent analyzes on each of the six BIS items. Mixed between-within subjects ANOVAs (group × time) were conducted in order to explore the impact of CPAP adherence at follow-up (adherence vs. non-adherence), insomnia diagnosis at baseline (chronic insomnia vs. not chronic insomnia), and OSA severity at baseline (AHI ≥ 30 vs. AHI < 30) on BIS scores (baseline vs. follow-up). Interaction effects were further explored using the pairwise *t* tests for simple effects. Mixed between-within ANOVAs were also conducted in order to explore the impact of follow-up time (< 20 vs ≥ 20 weeks) on BIS scores (baseline vs follow-up). We used median split to dichotomize the follow-up variable. Cohen’s *d* for paired values was calculated as a measure of effect size (*d* = M_1_-M_2_/SD pooled), considering *d* = 0.2 as small, *d* = 0.5 as moderate, and *d* = 0.8 as large effect sizes (Cohen, 1988). Multiple linear regressions with difference in BIS from baseline to follow-up as continuous dependent variable were performed, with sex, age, OSA severity, and CPAP adherence as covariates. Furthermore, we performed a binary (crude) logistic regression analysis with CPAP adherence (0 = adherent, 1 = non-adherent) as the dependent variable and BIS scores at baseline as predictor. An adjusted logistic regression analysis was also performed, with sex, age, and OSA severity at baseline as covariates. McNemar’s test (with continuity correction in 2 × 2 tables) was used to evaluate changes in the proportion of patients having chronic insomnia vs. patients who did not fulfill the criteria for chronic insomnia at baseline and at follow-up of CPAP treatment. Significance level was set to 0.05 for all analyses.

## Results

Descriptive statistics at baseline are presented in Table 1. Although follow-up was scheduled at around 3 months, the actual time for follow-up ranged from 6.0–51.9 weeks (mean 23.1 weeks, median 19.9 weeks). This was mostly due to patients re-scheduling their appointment. Mean CPAP adherence at follow-up was 4:35 h use per night (range 0:00 to 9:40 h, SD = 2:17). Mean AHI at follow-up, as measured by the CPAP device, was 2.7 (SD = 3.8, missing = 13).

**TABLE 1:** Descriptive statistics at baseline among Norwegian patients with obstructive sleep apnea.

Number of patients	442
Sex, males	74.4% (*n* = 329)
Age (mean ± SD)	54.9 ± 12.1
AHI (mean ± SD)	30.1 ± 21.1
AHI ≥ 30	38.9% (*n* = 172)
BIS total score (mean ± SD)	18.8 ± 9.8
Chronic insomnia	51.1% (*n* = 226)
Anxiety (missing = 11)	10.5% (*n* = 45)
Depression (missing = 12)	6.3% (*n* = 27)
BMI (mean ± SD) (missing = 9)	32.0 ± 6.1
Hypertension (missing = 9)	49.7% (*n* = 215)
Diabetes mellitus (missing = 10)	11.8% (*n* = 51)
Angina pectoris (missing = 27)	6.0% (*n* = 25)
Myocardial infarction (missing = 11)	7.7% (*n* = 33)
Stroke (missing = 22)	3.6% (*n* = 15)

*SD, standard deviation; AHI, apnea-hypopnea index; BIS, Bergen Insomnia Scale (0–42 scale); BMI, body mass index.*

*Anxiety and depression were measured with HADS, Hospital Anxiety and Depression Scale with 11 as cut-off.*

Table 2 shows results from separate analyses of items 1 to 6 in BIS. Notably, two items from BIS were reported on average more than 4 days per week at baseline: non-restorative sleep and dissatisfaction with sleep. There was a significant reduction in all items, *p* < 0.001, with a small effect size for the first three items and a moderate effect size for the last three items.

**TABLE 2:** Comparison of item 1–6 in BIS among 442 Norwegian patients with obstructive sleep apnea at baseline and follow-up (with CPAP).

	Baseline score	Follow-up score	Mean difference	Effect size	*T*-test
	
	Mean ± SD	Mean ± SD	Mean (95% CI)	Cohen’s *d*	*p*
1. SOL^a^ ≥ 30 min	1.98 ± 2.31	1.61 ± 2.09	0.38 (0.19–0.56)	0.19	** < 0.001**
2. WASO^b^	2.22 ± 2.36	1.59 ± 1.99	0.64 (0.44–0.84)	0.30	** < 0.001**
3. Early morning awakening	2.37 ± 2.34	1.95 ± 2.12	0.42 (0.22–0.62)	0.20	** < 0.001**
4. Non-restorative sleep	4.69 ± 2.26	3.04 ± 2.39	1.65 (1.42–1.89)	0.65	** < 0.001**
5. Daytime impairment	3.18 ± 2.48	1.81 ± 2.10	1.36 (1.13–1.60)	0.54	** < 0.001**
6. Dissatisfaction with sleep	4.33 ± 2.40	2.78 ± 2.35	1.55 (1.30–1.81)	0.58	** < 0.001**

*BIS, Bergen insomnia scale [the items are scored along an eight-point scale indicating the number of days per week (0–7 days)]; CPAP, continuous positive airway pressure; SD, standard deviation; CI, confidence interval. ^a^SOL, sleep onset latency. ^b^WASO, wake after sleep onset. Significant values are shown in bold.*

**FIGURE 2:**
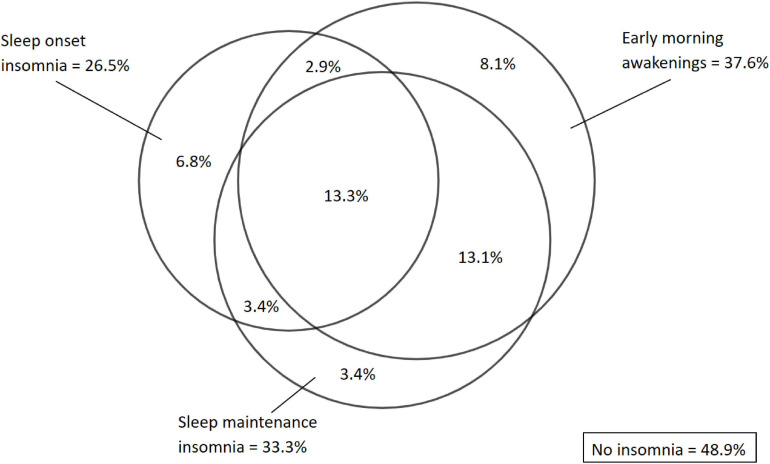
Prevalence and overlapping between insomnia subtypes at baseline among all patients with OSA (*n*= 442).

Figure 2 presents the overlapping between the insomnia subtypes. Among the 442 patients with OSA, 26.5% of the patients had sleep onset insomnia, 33.3% had sleep maintenance insomnia, and 8.1% had early morning awakenings. 13.3% had an overlap of all three insomnia subtypes, whereas isolated sleep onset insomnia, sleep maintenance insomnia and early morning awakenings presented in 6.8, 3.4, and 8.1% of the patients, respectively.

There was an overall decrease in insomnia symptoms as measured with BIS from baseline to follow-up, *p* < 0.001 (Table 3). Cohen’s *d* for repeated measures indicated a moderate to large effect size. Table 3 shows the results from the mixed between-within subjects ANOVAs conducted to assess the impact of CPAP adherence on changes in BIS scores. In terms of CPAP adherence, there was a significant group × time interaction effect [*F*(1,440) = 12.4, *p* < 0.001], indicating that the reduction in BIS scores was larger in the adherent group than in the non-adherent group (Table 3). Subsequent analyses of simple effects revealed a significant reduction in BIS scores for the adherent group with a large effect size (*p* < 0.001, Cohen’s *d* = 0.80), whereas the reduction in BIS scores for the non-adherent group was significant with a small to moderate effect size (*p* < 0.001, Cohen’s *d* = 0.43). There was a significant main effect of group, with higher BIS scores in the non-adherent group (*p* < 0.001) (Table 3). When analyzing patients with isolated sleep onset insomnia separately (*n* = 30), there was a significant decrease in BIS scores from baseline (mean = 20.7, SD = 5.2) to follow-up (mean = 13.9, SD = 8.2), *t*(29) = 5.07, *p* < 0.001. Cohen’s *d* (0.93) indicated a large effect size.

**TABLE 3:** Comparison of BIS scores at baseline and follow-up depending on adherence in 442 Norwegian patients with OSA treated with CPAP.

	Baseline BIS	Follow-up BIS	Mean difference	Effect size	*T*-test	ANOVA (*p-*value) (Group × time)		
						
	Mean ± SD	Mean ± SD	Mean (95% CI)	Cohen’s *d*	*p*	Time	Group	Interaction
All patients (*n* = 442)	18.78 ± 9.80	12.77 ± 9.87	6.00 (5.15–6.86)	0.65	** < 0.001**			
Adherent^a^ (*n* = 280)	17.89 ± 9.54	10.74 ± 8.87	7.16 (6.10–8.21)	0.80	** < 0.001**	**<0.001**	** < 0.001**	**<0.001**
Non-adherent^b^ (*n* = 162)	20.30 ± 10.08	16.30 ± 10.52	4.00 (2.58–5.44)	0.43	** < 0.001**			

*BIS, Bergen Insomnia Scale; OSA, obstructive sleep apnea; CPAP, continuous positive airway pressure; SD, standard deviation; CI, confidence interval. ^a^Adherent was defined as using the CPAP ≥ 4 h per night. ^b^Non-adherent was defined as using the CPAP < 4 h per night. Significant values are shown in bold.*

Table 4 shows the results from the mixed between-within subjects ANOVAs conducted to assess the impact of chronic insomnia and OSA severity on changes in BIS scores. There was a significant group × time interaction effect for the BIS score in terms of having chronic insomnia at baseline vs. not insomnia at baseline, *F*(1,440) = 38.1, *p* < 0.001 (Table 4). Scores were significantly reduced in both groups, with a large effect size in the group with chronic insomnia (*p* < 0.001, Cohen’s *d* = 0.90), and a small to moderate effect size in the group without chronic insomnia (*p* < 0.001, Cohen’s *d* = 0.45). In terms of OSA severity, there was a significant group × time interaction effect [*F*(1,440) = 4.1, *p* = 0.045], indicating that the reduction in BIS scores was larger in the group with severe OSA compared to the group with mild to moderate OSA (Table 4). Subsequent analyses for simple effects revealed a significant reduction in BIS scores in both groups with moderate effect sizes. There was a main effect of group, with higher BIS scores in the group with AHI < 30 (*p* = 0.003). In terms of follow-up time (< 20 vs ≥ 20 weeks), there was no significant group × time interaction effects for BIS, *F*(1,440) = 1.75, *p* = 0.186.

**TABLE 4:** Comparison of BIS scores at baseline and follow-up depending on insomnia diagnosis and severity of OSA in 442 Norwegian patients treated with CPAP.

	Baseline BIS	Follow-up BIS	Mean difference	Effect size	*T*-test	ANOVA (*p-*value) (Group × time)		
						
	Mean ± SD	Mean ± SD	Mean (95% CI)	Cohen’s *d*	*p*	Time	Group	Interaction
**Insomnia status**								
Chronic insomnia^a^ (*n* = 226)	25.51 ± 7.43	16.98 ± 10.16	8.53 (7.29–9.77)	0.90	**< 0.001**	**<0.001**	**< 0.001**	**<0.001**
Not insomnia (*n* = 216)	11.73 ± 6.44	8.37 ± 7.31	3.36 (2.27–4.44)	0.42	**< 0.001**			
**OSA severity**								
AHI ≥ 30^b^ (*n* = 172)	17.77 ± 9.44	10.67 ± 9.49	7.09 (5.64–8.56)	0.73	** < 0.001**	**<0.001**	**0.003**	**0.045**
AHI < 30^c^ (*n* = 270)	19.41 ± 9.99	14.11 ± 9.89	5.30 (4.25–6.35)	0.60	**< 0.001**			

*BIS, Bergen Insomnia Scale; OSA, obstructive sleep apnea; CPAP, continuous positive airway pressure; AHI, apnea-hypopnea index; SD, standard deviation; CI, confidence interval. ^a^Chronic insomnia was defined as scoring ≥ 3 days per week on at least one of the first three BIS items as well as ≥ 3 days per week on at least one of the two latter BIS items. ^b^AHI ≥ 30 was defined as severe obstructive sleep apnea. ^c^AHI < 30 was defined as mild to moderate obstructive sleep apnea. Significant values are shown in bold.*

Furthermore, multiple linear regressions with difference in BIS from baseline to follow-up (continuous parameter) as the dependent variable showed a positive association with CPAP adherence (hours) (B = 0.83, standard error (SE) = 0.19, beta = 0.21, *t* = 4.37, *p* < 0.001) when controlling for the influence of sex, age, and OSA severity. A negative association was seen with age (B = −0.08, SE = 0.04, beta = −0.10, *t* = −2.09, *p* = 0.037). There was no association with sex (B = 1.05, SE = 0.99, beta = 0.05, *t* = 1.06, *p* = 0.29) or OSA severity (B = 0.03, SE = 0.02, beta = 0.06, *t* = 1.30, *p* = 0.19). In the logistic regression analysis, we found that higher BIS scores at baseline predicted non-adherence to CPAP, B = 0.03, SE = 0.1, wald = 6.14, Exp (β) = 1.03, 95% CI = 1.01–1.05, *p* = 0.013. The association remained the same when adjusting for sex, age, and OSA severity (OR = 1.03, 95% CI = 1.01–1.05, *p* = 0.012).

The proportion of patients with chronic insomnia was significantly reduced from 51.1% at baseline to 33.0% at follow-up (*p* < 0.001). A McNemar’s test showed that 31.2% (*n* = 138) changed category at follow-up of CPAP treatment. Among these, significantly more participants (24.7%, *n* = 109) changed from chronic insomnia to not having insomnia, than from not having insomnia to chronic insomnia (6.6%, *n* = 29), χ^2^ (1, *n* = 442) = 45.23, *p* < 0.001 (two sided).

## Discussion

We found a significant decrease in symptoms of insomnia at follow-up compared to baseline. The adherent group had a significantly larger reduction in insomnia symptoms, although both groups showed clearly improved sleep. Secondly, there was a significant reduction in the prevalence of patients who fulfilled the diagnosis of chronic insomnia at follow-up compared to baseline. These findings are in agreement with our initial hypothesis of a reduction in both insomnia symptoms and prevalence of chronic insomnia at follow-up after starting CPAP treatment, and that the improvement would depend on CPAP adherence.

As expected, we found a clearly higher prevalence of chronic insomnia (51.1%) at baseline among these patients with OSA, compared to means of 8–20% in the general Norwegian population (Pallesen et al., 2014; Uhlig et al., 2014; Bjorvatn et al., 2018b). The current prevalence is in accordance with previous studies of comorbid insomnia among patients with OSA (Krakow et al., 2001; Björnsdóttir et al., 2012). In line with previous studies (Chung, 2005; Björnsdóttir et al., 2013), wake after sleep onset (WASO) and early morning awakening (EMA) symptoms were more commonly reported than sleep onset latency (SOL) symptoms. However, sleep maintenance insomnia was found in 33.3% of the patients compared to 59.4% in a similar Icelandic cohort study, and isolated sleep maintenance insomnia was found in only 3.4 vs. 33.0% of the patients from Iceland (Björnsdóttir et al., 2013). The reason for these differences may be that insomnia subtypes were measured differently in the two studies. The Icelandic study used the Basic Nordic Sleep Questionnaire and looked at frequency of awakenings, whereas BIS looks at number of days a week where the patient has been awake for more than 30 min between periods of sleep. In addition, the Icelandic study did not include questions about daytime impairment. To diagnose insomnia in accordance with DSM-5 and ICSD-3, some sort of daytime impairment or dissatisfaction with sleep has to be present (American Psychiatric Association, 2013; American Academy of Sleep Medicine, 2014). Furthermore, the insomnia symptoms should not be better explained by other sleep disorders (American Academy of Sleep Medicine, 2014). It is well-known that OSA may disturb sleep by increasing the number of nightly awakenings (Krakow et al., 2001; Smith et al., 2004). Future research should therefore aim to develop a validated scale measuring insomnia symptoms and severity in patients with COMISA focusing on non-overlapping symptoms in addition to daytime impairments and nocturnal symptoms.

Previous studies have shown that treatment with CPAP is effective in reducing symptoms of insomnia (Björnsdóttir et al., 2013; Glidewell et al., 2014). The present findings provide evidence that CPAP has a positive impact of both insomnia symptoms and the prevalence of chronic insomnia. Sweetman et al. (2019a) point out that overlapping symptoms in patients with COMISA should warrant caution when interpreting effect of CPAP on insomnia symptoms, as a reported reduction of daytime impairments (most likely due to reduced apneas) may be interpreted as an overall reduction of insomnia severity score even in the absence of improved nocturnal symptoms. Although sleep maintenance symptoms may be caused by OSA, sleep onset problems are less likely to improve with CPAP (Björnsdóttir et al., 2013). However, our results showed a significant decrease in all six items measured with BIS, and the overall decrease in insomnia symptoms remained the same also when only patients with sleep onset problems were included in the analyses.

There was a significant group difference between adherent and non-adherent patients in terms of higher insomnia scores in the non-adherent group. Furthermore, we found that higher BIS scores at baseline predicted non-adherence to CPAP. This is in accordance with previous studies finding lower adherence to CPAP in patients with COMISA compared to OSA alone (Wickwire et al., 2010; Eysteinsdottir et al., 2017). Many factors, including having to wear a CPAP mask, leakage, and noise from the machine may leave patients with comorbid insomnia less tolerant to CPAP. Although there was a significant improvement in terms of reduced insomnia scores in both the adherent and the non-adherent group, the effect size was clearly higher in the adherent group. This is also in line with previous studies (Glidewell et al., 2014). Hence, in clinics with focus on treating OSA, this finding underlines the importance of follow-up consultations provided by trained healthcare providers to ensure CPAP adherence. Furthermore, OSA patients should be routinely evaluated for insomnia symptoms and be considered for specific insomnia treatment (such as CBTi), especially if patients have poor adherence to CPAP or if the insomnia symptoms are not improved after CPAP treatment (Sweetman A. M. et al., 2017). The evidence for whether CBTi can improve adherence to CPAP is not entirely clear (Ong et al., 2020b). One recent randomized controlled trial reported that CBTi prior to CPAP treatment improved both CPAP adherence and insomnia symptoms (Sweetman et al., 2019b), whereas another recent randomized controlled trial did not find significantly improved adherence (Ong et al., 2020a). The contradicting findings might be due to different inclusion criteria (moderate to severe OSA vs. mild OSA), and more research is warranted.

As expected, the effect size was larger when patients with chronic insomnia vs. not chronic insomnia were analyzed separately, although both groups had a significant reduction in insomnia symptoms at follow-up. However, this could also partly be due to “regression to the mean” (Barnett et al., 2005), a statistically phenomenon in which patients with high (or low) scores at baseline are more likely to get closer to the mean if measured a second time, independently of CPAP adherence. Sweetman A. et al. (2017) suggest that patients with COMISA should be treated with CBTi prior to CPAP treatment to improve outcome measures both in terms of CPAP adherence and insomnia symptoms. In the present study, however, 48.2% (109/226) of the patients having chronic insomnia at baseline according to DSM-5 criteria did not have insomnia at follow-up. It is possible that some of these patients experienced insomnia as a result of OSA manifestations, and in that case the insomnia symptoms are less likely to improve with CBTi (Björnsdóttir et al., 2013; Glidewell et al., 2014). This further supports the notion that patients referred to a sleep clinic because of OSA may benefit from CPAP treatment in terms of reducing both insomnia symptoms and prevalence of insomnia. However, 51.8% of the patients with chronic insomnia at baseline had persistent insomnia at follow-up, and if the treatment of OSA does not result in improvement of insomnia symptoms at follow-up, these patients should be recommended CBTi, as several studies have reported that COMISA patients receiving both CBTi and CPAP therapy have significantly greater improvement of global insomnia symptoms after 6 months (Sweetman et al., 2019b; Ong et al., 2020a; Alessi et al., 2021). Our data implicate that referral for combined CBTi and CPAP therapy may be appropriate for at least half of the patients who do not respond to CPAP alone. Although many patients reported improved sleep and less insomnia symptoms after treatment with CPAP, 13.4% (29/216) of the patients who did not have chronic insomnia at baseline fulfilled the diagnostic criteria at follow-up. It is possible that problems with CPAP, such as noise from the machine, leakage and problems wearing the mask might have induced chronic insomnia in these patients.

In a cross-sectional study among OSA patients, Bjorvatn et al. (2015) found that symptoms of insomnia were negatively associated with OSA severity, also after adjusting for relevant confounders such as sex, age, smoking, alcohol consumption, and obesity. In the present study, there was a significant group difference between patients with mild to moderate OSA and patients with severe OSA in terms of lower BIS scores among patients with severe OSA. The significant (group × time) interaction between OSA severity and insomnia scores indicated that although patients with severe OSA had less insomnia, they showed a greater reduction in insomnia symptoms and might benefit more from CPAP than patients with mild to moderate OSA. This finding is in accordance with Glidewell et al., who found that improvement of insomnia was linked to more severe OSA (Glidewell et al., 2014). The authors suggested that the effect of CPAP on insomnia may be mediated by OSA severity. However, there was no association with OSA severity when entered as covariate in multiple linear regression with difference in BIS from baseline to follow-up as dependent variable. There was a positive association with CPAP adherence, and this indicates that adherence to CPAP is the most important factor for reduction in insomnia symptoms among these patients.

Recently, Bjorvatn et al. (2018a) performed a randomized controlled trial among 164 patients with COMISA who were initiating CPAP and found that treating their insomnia with a self-help book did not improve sleep more than sleep hygiene advice. However, both groups showed a significant reduction in insomnia symptoms (measured with BIS and Insomnia Severity Index). The authors suggested that the improvement in sleep was most likely explained by the CPAP treatment, and this notion is further supported by the current study.

There are several strengths and limitations of the present study that should be noted. The relatively large sample size provided statistical power to the analyses. Furthermore, the insomnia questionnaire is well validated. The scale has shown acceptable test-retest reliability, as well as good convergent and discriminative validity in relation both to other self-report measures and to polysomnographic data (Pallesen et al., 2008). In addition, this is a clinical sample with patients referred to a university hospital for suspicion of OSA from a large geographic area in Western Norway. Thus, we believe it is representative for the overall OSA population and their different comorbidities. One possible limitation was that the time frame in BIS was changed from complaints during the past month to the past 3 months in 2018, due to the new diagnostic criteria in DSM-5. Thus, 369 of the 442 patients answered BIS regarding the past month. However, a study from Morin et al. (2009) showed that the risk of still having insomnia after 1 year in patients having insomnia for more than 1 month is 74%. These results suggest that insomnia for most people is a chronic disorder, and we do not believe that the fact that most patients answered BIS regarding the past month introduced a problem in the interpretation of the present results. The low percentage of patients with isolated sleep maintenance insomnia (3.4%) did not provide us enough statistical power to conduct separate analyses to compare this insomnia subtype to the sleep onset and early morning awakening insomnia subtypes, as done in previous studies (Wickwire et al., 2010; Björnsdóttir et al., 2013). Another limitation was that polygraphy sleep testing commonly underestimates the number of obstructive respiratory events per hour compared to polysomnography, as polygraphy monitors the number of apneas/hypopneas per hour monitoring time rather than per total sleep time (American Academy of Sleep Medicine, 2014). Also, a limitation was that we did not include a control group randomized to not receiving CPAP therapy in this study. We have no data about whether these patients received other kinds of treatment in the follow-up period, such as sedative-hypnotics or CBTi, and this was a limitation. Furthermore, although follow-up was scheduled at 3 months, the time interval between baseline and follow-up varied considerably. This was mostly due to patients re-scheduling their appointment. We excluded patients attending follow-up in less than 1 month and more than 1 year after baseline, and the statistical analyses showed no significant interaction between follow-up time (<20 vs. ≥20 weeks) and decrease in BIS scores. The CPAP device reports an average use over the last 90 nights. However, we do not know how many days of use each patient have, and this was a limitation in our study.

## Conclusion

We found a decrease in symptoms of insomnia as measured with Bergen Insomnia Scale from baseline to follow-up of CPAP treatment. The improvement was significant in both adherence groups, but the degree of improvement was larger among patients adherent to CPAP. Furthermore, there was a significant reduction in patients who fulfilled the diagnosis of chronic insomnia at follow-up compared to baseline. This suggests that CPAP may be effective in reducing both insomnia symptoms and the prevalence of chronic insomnia in some OSA patients.

## Data Availability Statement

Data cannot be released into a publicly accessible repository due to concerns regarding participant anonymity as raised by the Regional Committee for Medical and Health Research Ethics of Western Norway (REC-West).

## Ethics Statement

The studies involving human participants were reviewed and approved by The Regional Committee for Medical and Health Research Ethics, Health Region West (REC 2018/337). The patients/participants provided their written informed consent to participate in this study.

## Author Contributions

All authors contributed substantially to the conception/design of the work, or the analysis or interpretation of the data. Furthermore, all authors drafted or revised the manuscript, and approved the final version of the manuscript.

## Conflict of Interest

The authors declare that the research was conducted in the absence of any commercial or financial relationships that could be construed as a potential conflict of interest.
